# Evaluation of Effectiveness of a Community-Based Intervention for Control of Dengue Virus Vector, Ouagadougou, Burkina Faso

**DOI:** 10.3201/eid2410.180069

**Published:** 2018-10

**Authors:** Samiratou Ouédraogo, Tarik Benmarhnia, Emmanuel Bonnet, Paul-André Somé, Ahmed S. Barro, Yamba Kafando, Diloma Dieudonné Soma, Roch K. Dabiré, Diane Saré, Florence Fournet, Valéry Ridde

**Affiliations:** University of Montreal Public Health Research Institute, Montreal, Canada (S. Ouédraogo, D. Saré, V. Ridde);; University of California, San Diego, California, USA (T. Benmarhnia);; Institut de Recherche pour le Développement, Bondy, France (E. Bonnet);; Action Gouvernance-Intégration-Renforcement, Ouagadougou, Burkina Faso (P.A. Somé, A.S. Barro, Y. Kafando);; Institut de Recherche en Science de la Santé, Bobo-Dioulasso, Burkina Faso (D.D. Soma, R.K. Dabiré);; Institut de Recherche pour le Développement, Montpellier, France (F. Fournet);; Institut de Recherche pour le Développement, Paris (V. Ridde);; Institut National de la Santé et de la Recherche Médicale, Paris (V. Ridde)

**Keywords:** dengue, vector-borne disease, arbovirus, Aedes aegypti, mosquitoes, community-based, intervention, effectiveness, sub-Saharan Africa, Burkina Faso, viruses

## Abstract

We evaluated the effectiveness of a community-based intervention for dengue vector control in Ouagadougou, the capital city of Burkina Faso. Households in the intervention (n = 287) and control (n = 289) neighborhoods were randomly sampled and the outcomes collected before the intervention (October 2015) and after the intervention (October 2016). The intervention reduced residents’ exposure to dengue vector bites (vector saliva biomarker difference −0.08 [95% CI −0.11 to −0.04]). The pupae index declined in the intervention neighborhood (from 162.14 to 99.03) and increased in the control neighborhood (from 218.72 to 255.67). Residents in the intervention neighborhood were less likely to associate dengue with malaria (risk ratio 0.70 [95% CI 0.58–0.84]) and had increased knowledge about dengue symptoms (risk ratio 1.44 [95% CI 1.22–1.69]). Our study showed that well-planned, evidence/community-based interventions that control exposure to dengue vectors are feasible and effective in urban settings in Africa that have limited resources.

Since 2010, dengue outbreaks have been detected repeatedly in several countries in sub-Saharan Africa (*1*–*4*). The resurgence of dengue outbreaks in the region might be explained by factors such as urbanization, globalization, lack of effective mosquito control, and climate change (*5*,*6*). Dengue virus (DENV) belongs to the *Flaviviridae* family and has 4 serotypes (DENV-1 to DENV-4) (*7*) that cause human disease through transmission by infected female mosquitoes, mainly *Aedes* mosquitoes. These mosquitoes have fully adapted to urban settings, where crowded human populations live in close proximity to large mosquito populations (*8*). Although DENV-2 has been reported most frequently, all 4 DENV serotypes are circulating in Africa (*9*). *Ae. aegypti* was found to be the main species in urban settings (*10*). Future climate projections indicate considerable potential for shifting establishment of *Ae. aegypti* mosquitoes in all regions of the world and especially in Africa (*11*). However, dengue continues to be a neglected disease in this region, often eclipsed by the substantial burden of malaria (*12*). Dengue infection is usually not included among the differential diagnoses of acute febrile illness (*13*).

The World Health Organization (WHO) has stated that effective vector control measures are critical to achieving and sustaining reduction of disease attributable to dengue (*14*). Common dengue vector control measures, which are typically community-driven in tandem with health promotion campaigns, include use of insecticide-treated materials (*15*) or water storage tanks (*16*) and elimination of breeding sites or use of larvicides (*17*). The environment can be modified to deprive mosquito vectors of favorable breeding sites. A growing body of evidence indicates that changes in these conditions have led to alterations in the prevalence, spread, geographic range, and control of many infections transmitted by these vectors (*14*). Many community-level interventions have been conducted in Asia and Latin America (*18*); overall, the results suggest that these interventions led to a reduction of vector densities. However, we did not find any reports about community-based interventions (CBIs) aimed at controlling the dengue vector in Africa, nor did Bowman et al. (*18*) in a recent systematic review.

Our study describes an evaluation of the effectiveness of a CBI for dengue vector control in a neighborhood of Ouagadougou, the capital city of Burkina Faso (*19*). We chose this city because dengue outbreaks were detected in Ouagadougou in 2013 (*12*). DENV-2 has been endemic for more than 30 years in the country, and 3 serotypes (DENV-1, DENV-2, and DENV-3) have been identified (*12*,*20*,*21*), leading to the occurrence of more severe cases often not captured by the relatively weak surveillance system, which has resulted in underreporting (*22*) and a lack of national coordinate response activities.

## Population and Methods

### Study Site and Participants

The study was conducted in 2 comparable neighborhoods of Ouagadougou, Tampouy and Juvenat, selected from a total of 5 areas in the city (Technical Appendix Figure 1). Both neighborhoods’ socio-economic profiles are highly diverse (*23*) and include wealthy households in modern concrete individual houses with running water and electricity, households with a modest standard of living, and poor people living in fairly small houses in the same compound, sometimes without basic amenities. We defined a household as a person or a group of persons with the same head of household, living in a housing unit, who provide themselves with food or other essentials for living; in Burkina Faso, 1, 2, or more households living in different housing units sometimes share the same compound. 

**Figure 1:**
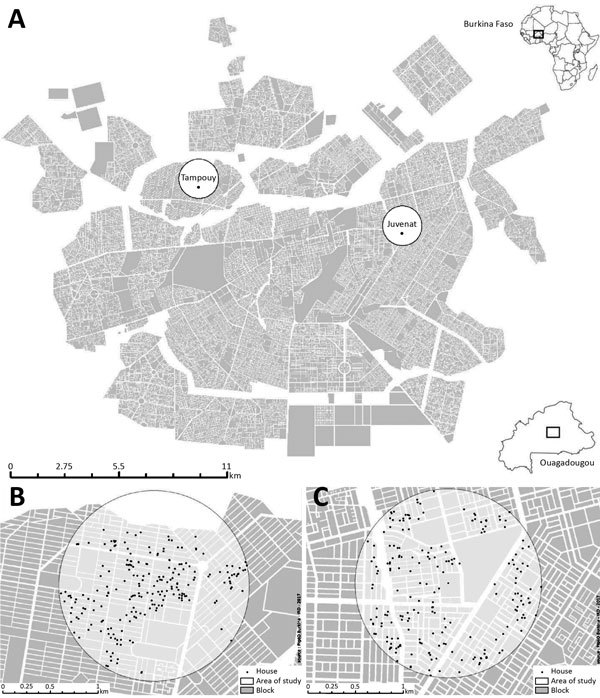
Intervention and control areas for an evaluation of a community-based intervention for dengue vector control conducted in Ouagadougou, Burkina Faso, June–October 2016. A) Ouagadougou overview; inset shows location of Ouagadougou in Burkina Faso. B) Tampouy (intervention neighborhood). C) Juvenat (control neighborhood).

Tampouy (Figure 1), located in the northwest of Ouagadougou, was randomly chosen to receive the intervention, whereas Juvenat, on the east side of the city, was selected as the control neighborhood. In 2015, we estimated that 4,264 households were located within a 1-km radius around the primary healthcare center in Tampouy. In the similarly delimited area in the control neighborhood of Juvenat, we identified 3,294 households. We chose a 1-km radius to reduce the probability of contamination between the control and intervention areas while also having a sufficient number of households for the study. The number of households in the area were estimated by using data from Burkina Faso’s National Institute of Statistics and Demography and data collected through very high spatial resolution satellite imagery, which also enabled the distinction of dwellings from other types of buildings. To measure the study outcomes, we used the geographic coordinates of households to randomly sample without replacement 287 households in Tampouy and 289 households in Juvenat. In this study, we considered a compound as a delimited living space where >1 household was found.

### Intervention Design

The CBI occurred during June–early October 2016. Because this period is the rainy season, it is also the peak dengue transmission period (*24*).

We used an ecohealth intervention approach that consists of a pesticide-free dengue vector control (*25*). The theoretical approach to creating the communication materials is described in Technical Appendix Figure 2. The intervention neighborhood received a behavior change intervention structured around 3 components (Technical Appendix Table 1). The intervention design is based on selected effective CBIs in controlling dengue vector (*25*–*28*) through a participatory process with community leaders. Selected community members, leaders, and a community theatrical troupe received training on dengue prevention. These community members then organized community activities and served as educators.

Key messages addressed WHO recommendations for identifying *Ae. aegypti* mosquito breeding sites and dengue transmission, symptoms, management, and prevention (*14*). Education materials created through the participatory process included posters created by workshop participants, which were then professionally drawn by a local artist, and a theater piece illustrating the key messages: 1) how dengue is transmitted, its symptoms, and how it differs from malaria; 2) timely use of health services; and 3) how to prevent dengue and strategies to identify and control *Ae. aegypti* mosquito breeding sites.

Community leaders were identified by community members. They included those responsible for places of worship (e.g., churches and mosques), representatives of community associations, and community health workers collaborating with the primary healthcare center. Participation in the intervention was voluntary. Community leaders invited members to participate in the intervention, and an announcer was hired to travel with a loudspeaker along every street in the intervention neighborhood to invite everyone. Interested persons attended communication and education activities, including community theater, which involved a play, interaction of the actors with the audience, and a question and answer session, as well as community clean-up activities conducted in the public spaces. The intervention also included door-to-door visits, school education, and self-awareness assessment sessions that involved education with messages intended to raise student awareness of dengue and provide information on the disease by using posters. These events were followed by a poster drawing competition among all students, illustrating the key messages they had learned about dengue.

In the control area, no communication activities were carried out for dengue awareness and control. The risk of cross-contamination between the 2 sites was low because the control area was located >12 km from the intervention area.

All intervention activities were coordinated by 5 researchers with experience in implementation of CBIs and 5 experienced entomologists and conducted by 17 community members and theater actors with experience conducting CBIs, recruited and trained for the intervention, and 7 community representatives (e.g., traditional chiefs, religious leaders, and local association heads) for a peer review and follow-up of the activities. An evaluation of the intervention design and implementation processes showed that most of the activities had been carried out as planned with only minor modifications (Technical Appendix Table 1).

### Outcomes

Before the intervention, in late October 2015, we performed a baseline data collection in the control and intervention neighborhoods. We collected the same data in late October 2016 in both neighborhoods (after the intervention and during the peak dengue transmission period for 2016) to assess the effectiveness of the intervention. The primary outcomes (serologic and entomologic data) were collected at the compound level and the secondary outcomes (data on knowledge, attitudes, and practices) at the household level, given that in our study neighborhood we found >2 households living in the same compound (*29*). The residents of the compound are exposed to the same population of mosquitoes. However, depending on their age, education level, and sanitation habits, individual households might have different levels of knowledge and attitudes about dengue and its prevention.

#### Primary Outcomes (Continuous Variables)

##### Immunologic Biomarkers

Evidence of compound residents’ exposure to *Ae. aegypti* mosquito bites was used to measure the population’s exposure to mosquito bites. In each compound, 2 residents (1 child and 1 adult) present at the time of data collection were randomly sampled to provide blood drops for an *Ae. aegypti* mosquito saliva biomarker test. ELISA was performed on these standardized dried blood spots, and results were expressed as ΔOD (optical density), defined as the level of IgG to Nterm-34 kDa peptide. These ΔOD values were calculated according to the formula ΔOD = ODx – 2ODn, where ODx represents the mean of individual OD values in antigen wells and ODn the OD value in a well with no antigen (*30*). The measurement of immunologic response to *Ae. aegypti* mosquito saliva in human populations has been documented as a relevant tool to assess a host’s level of exposure to *Ae. aegypti* mosquito bites and the risk for vectorborne disease (*31*).

##### Entomologic Data

In the compound where blood samples were collected, interviewers were asked to identify all *Ae. aegypti* mosquito breeding sites with water and to collect and count all the larvae and pupae from the containers. The water was poured out of the containers only at the endline survey, and residents were advised to avoid these kinds of containers. Standard entomologic indices included the *Ae. aegypti* mosquito house index (compounds with larvae or pupae × 100 compounds examined), container index (containers with larvae or pupae × 100 containers examined), Breteau index (containers with larvae or pupae per 100 compounds examined), and pupae index (pupae per 100 compounds examined) (*31*). These indices were generated at the neighborhood level.

#### Secondary Outcomes

The secondary outcomes were self-reported knowledge, attitudes, and practices (categoric variables), collected during a face-to-face interview with an adult household respondent.

Knowledge about dengue was assessed by asking “Can you list diseases that include fever as a symptom?”; “Have you ever heard of dengue?”; “Is dengue a form of malaria?”; and “Is dengue dangerous?” Knowledge about dengue’s mode of transmission was assessed by asking “Is dengue transmitted by the same mosquito as malaria?” Attitudes and practices for preventing dengue fever and diseases causing fever were assessed by asking “Do you store water in containers?”; “Do you cover your water containers?”; and “Do you use bed nets?”

All data were collected by trained interviewers who did not participate in the intervention. Each neighborhood had its own interview team at baseline and endline, and the interviewers’ work was supervised by the research team. The questionnaire was administered using the free Open Data Kit software (https://opendatakit.org).

Verbal consent was obtained from the respondents to the household questionnaire and from those who provided a blood sample. For children providing samples, at least 1 parent provided consent.

### Statistical Analysis

We used a propensity score (PS) stratification approach to estimate the effect of the intervention on the outcomes of interest while ensuring that covariate balance was achieved between intervention and control groups (*32*). After estimating the PS for the control and the intervention groups (Technical Appendix Figure 3), we excluded compounds and households with a PS outside of the overlapping intervention–control zone of the PS distribution. To obtain the optimal stratification setting while keeping a good covariate balance, we stratified the distribution of PS by using a 5-quantile approach, as recommended previously (*32*). This approach enabled exchangeability between intervention and control groups by ensuring that neighborhoods within a specific propensity score strata were compared. We modeled the propensity of receiving the intervention by using a logistic regression model that included the following covariates as independent variables: the number of households; sets of bedding in the compound and residents in the compound; the status of the person who provided the blood sample (adult or child); the household or households wealth index quintile (1 being the poorest and 5 the richest); and the questionnaire respondent characteristics such as status (head of the household, lady of the household, or other responsible adult), sex (male or female), and a variable that specified the respondent’s self-reported reading ability (cannot read, can read, or can read with difficulties). 

To estimate the effect of the intervention on the changes in outcomes in each specific household, we used linear regression models (with a fixed effect for the PS strata) for continuous outcomes. For binary outcomes, we used Poisson regression models with robust variance (with a fixed effect for the PS strata) (*33*). Using a modified Poisson model (i.e., with robust variance) has been shown to be a good alternative to logistic regression, especially when the outcome prevalence is not small. The Poisson model also gives the risk for the exposed group (not the odds ratio, as in logistic regression). Analyses were conducted by using Stata 14.2 (StataCorp LLC, College Station, TX, USA).

Serologic analysis was performed by using GraphPad Prism5 software (San Diego, CA, USA) (*30*). The descriptions of the covariates, data analysis, and results are provided in Technical Appendix Figure 4.

## Results

### Baseline and Endline Data

**Figure 2:**
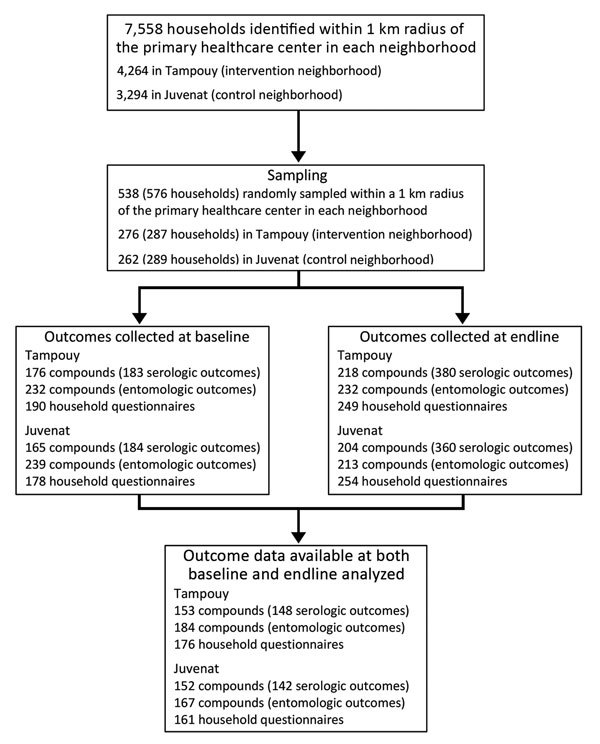
Flowchart for identification of compounds and households for a community-based intervention for dengue vector control conducted in Ouagadougou, Burkina Faso, June–October 2016.

The number of compounds and households randomly sampled, those who completed the study at baseline and endline, and the numbers included in the data analyses are detailed in Figure 2. We summarized the characteristics of the compounds at baseline (2015) and endline (2016) (Technical Appendix Table 2). In the control neighborhood, among households for which responses to the questions were obtained at baseline, heads of household were more likely to be the respondents to the questionnaire (88/161 [54.66%]) than in the intervention neighborhood (31/176 [17.61%]) and were less likely to be female (78/158 [49.37%]) than in the intervention neighborhood (130/173 [75.14%]). The respondents had lower reading ability in the intervention neighborhood, 108 (62.79%) of 172 respondents could read, compared with 133 (84.71%) of 157 in the control neighborhood.

### Outcomes and Estimation

We summarized residents’ immunologic response to *Ae. aegypti* mosquito bites in the intervention and control neighborhoods (Technical Appendix Table 3). The raw propensity score mean (± SD) was 0.50 (± 0.13) for all observations. For intervention neighborhood observations, PS was 0.53 (± 0.10); for control neighborhood observations, PS was 0.47 (± 0.15). After stratification, the within-strata differences in the PSs between intervention and control observations ranged from 0.01 to 0.03.

At baseline, residents showed higher exposure to *Ae. aegypti* mosquito bites in the intervention neighborhood (∆OD mean [± SD] 0.17 [± 0.10]) than the control neighborhood (∆OD 0.13 [± 0.06]). At endline, residents from the intervention neighborhood showed lower exposure to *Ae. aegypti* mosquito bites (∆OD 0.18 [± 0.08]) than the control neighborhood (∆OD 0.20 [± 0.12]). The regression analysis on residents’ immunologic response showed that the intervention reduced exposure to *Ae. aegypti* mosquito bites (coefficient −0.08 [95% CI −0.11 to −0.04]).

**Figure 3:**
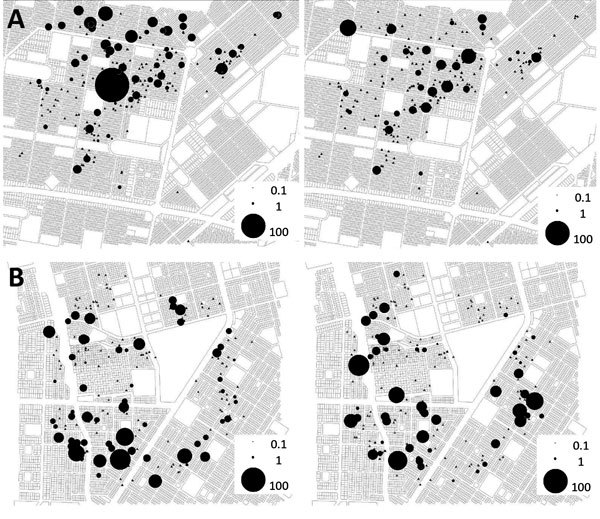
*Aedes aegypti* larvae and pupae per resident (black dots) in the compounds of (A) intervention neighborhood (Tampouy) and (B) control neighborhood (Juvenat) at baseline (left) and endline (right) of an evaluation of a community-based intervention for dengue vector control conducted in Ouagadougou, Burkina Faso, June–October 2016.

In Tampouy, the container index decreased in the intervention neighborhood (from 17.56% to 14.43%) and increased in the control neighborhood (30.41% to 35.91%), similar to what was observed for the pupae index (decreasing from 162.14 to 99.03 in the intervention and increasing from 218.72 to 255.67 in the control neighborhood). A greater decrease was observed in the house index in the intervention neighborhood (from 32.04% to 21.36%) compared with the control neighborhood (from 33.00% to 31.53%) as well as in the Breteau index (from 40.77% to 27.67% in the intervention neighborhood compared with 54.19% to 48.28% in the control neighborhood (Figure 3; Technical Appendix Table 3). However, the regression models did not show an effect of the intervention on the absolute number of *Ae. aegypti* mosquito breeding sites or on the number of preimaginal stages of vector (larvae and pupae) at the compound level.

The households that received the intervention increased their knowledge of dengue (risk ratio [RR] 1.13 [95% CI 1.01–1.27]) and disease symptoms (RR 1.44 [95% CI 1.22–1.69]) and were less likely to associate dengue with malaria (RR 0.70 [95% CI 0.58–0.84]). Respondents self-reported that they had increased their actions against mosquitoes (RR 1.42 [95% CI 1.29–1.57]) and used more bed nets (RR 1.31 [95% CI 1.22–1.42]).

## Discussion

Our study assessed the effectiveness of a CBI for dengue vector control in Ouagadougou, Burkina Faso. This evidence-based intervention was developed with local stakeholders, adapted to the community, and implemented following an ecohealth approach (*26*).

In Tampouy, the intervention reduced residents’ exposure to *Ae. aegypti* mosquito bites and container and pupae indices, whereas these indices increased in Juvenat, the control neighborhood. House and Breteau indices also had a greater reduction in the intervention neighborhood. Knowledge about dengue was very limited at baseline in both the intervention and control neighborhoods. Respondents in the intervention neighborhood had increased knowledge about dengue and actions to control mosquitoes, a first step in the process of dengue vector control activities. In the control neighborhood, limited knowledge of dengue transmission, prevention, and treatment resulted in poorer protective practices against dengue vector. These results are in line with those from previous studies performed in Asia and Latin America (*26*–*28*,*34*,*35*).

In both the intervention and control neighborhoods, the entomologic indices were high at baseline, which might be the case for the entire city. Residents might not be aware of the conditions or factors that can exacerbate the presence of dengue vectors. Moreover, a real need exists to characterize *Ae. aegypti* mosquito breeding sites in Ouagadougou so they can be specifically targeted through education and vector control activities. Entomologic studies are also needed to clarify the ecologic aspects of the *Ae. aegypti* mosquito; strengthened disease surveillance is also needed because persons can be bitten by mosquitoes outside of the home. An integrated surveillance system (i.e., addressing epidemiology and entomology) allows for data triangulation, which should lead to better vector control planning.

We did not find an effect of the intervention on the number of *Ae. aegypti* breeding sites or the number of larvae and pupae found in compounds. Water stored for a long time became stagnant and a potential *Ae. aegypti* mosquito breeding site. Residents in the intervention neighborhood might have adopted measures to protect themselves from *Ae. aegypti* mosquito bites, which is confirmed by the reduction in the immunologic biomarkers; however, they might have developed the habit of pouring out or covering water containers. Moreover, the interviewers knew the intervention status of the neighborhoods, which could have led to potential reporting bias and might be seen as a limitation of the study. However, the results of the serologic biomarkers and the household questionnaires showed that any potential bias was minimal.

Persons’ health beliefs and their dengue-related knowledge, attitudes, and practices are likely to shape their healthcare practices and behaviors (*36*). The success of dengue prevention and mosquito control efforts in the community relies on the effectiveness of initiatives to educate the public about dengue and how it spreads, how the general public can control *Ae. aegypti* mosquito breeding sites, and how to improve household environmental sanitation through sustained modification of human behavior (*37*).

According to Stahl et al. (*38*), preventing dengue outbreaks is much cheaper than paying for the consequences of an outbreak. Burkina Faso is experiencing an alarming increase in dengue cases and the dengue vector population during raining seasons. The spread of the vector is associated with climate change, globalization, and rapid urbanization (*39*); however, many other major diseases can also be transmitted by the mosquito vectors of dengue (e.g., yellow fever, chikungunya, and Zika) (*5*). WHO has recommended that any country in the dengue belt with *Ae. aegypti* mosquitoes should be vigilant about the spread of Zika virus (*40*). Understanding which interventions are effective in what context is needed to prevent new diseases that could be established with competent vectors and to control current diseases. Now that the favorable environmental conditions for Zika vector spread have been confirmed to exist in Africa, complacency is not an option (*41*).

Considerable enthusiasm exists for novel vector control approaches to prevent diseases transmitted by *Ae. aegypti* mosquitoes, including 1) release of mosquitoes infected with a strain of *Wolbachia* spp. bacteria; 2) release of large numbers of sterile male vectors; 3) use of mosquitoes engineered to carry a lethal gene; and 4) use of pyriproxyfen, a powerful synthetic analog of mosquito juvenile hormone (*42*). An effective prevention and control strategy against *Aedes* mosquito–borne diseases in tropical urban settings includes a strong community effort in social mobilization and communication, along with use of new technologies that combine enhanced mosquito control with effective vaccines and improved diagnosis and clinical management, including the use of antivirals and therapeutic antibodies (*43*). 

In sub-Saharan Africa, the promotion of health literacy is critical to active and informed participation in health promotion and disease prevention (*44*); it is one component that can facilitate or be a barrier to a health education and communication intervention for dengue vector control. Public health authorities should sustain their education and communication efforts and the budget for such efforts not only when an outbreak is ongoing. Communities must be reminded of when to carry out the actions, how to properly carry out the recommended behaviors, and what the benefits are to carrying them out. To achieve sustained behavior changes in dengue vector control, continuous communication and interaction between governmental agencies and the communities is essential.

Technical Appendix Additional information on evaluation of the effectiveness of a community-based intervention for the control of dengue virus vector in Ouagadougou, Burkina Faso.
