# Supplementary material for: Evaluation of Effectiveness of a Community-Based Intervention for Control of Dengue Virus Vector, Ouagadougou, Burkina Faso
Source: Emerg Infect Dis. 2018 Oct;24(10):1859–67. doi: 10.3201/eid2410.180069 (PMC6154160; doi:10.3201/eid2410.180069)
Supplement: Technical Appendix — Additional information on evaluation of the effectiveness of a community-based intervention for the control of dengue virus vector in Ouagadougou, Burkina Faso. [file 18-0069-Techapp-s1.pdf]

Article DOI: https://doi.org/10.3201/eid2410.180069

Evaluation of Effectiveness of a Community-Based Intervention for Control of Dengue Virus Vector, Ouagadougou, Burkina Faso

Technical Appendix

Technical Appendix Table 1. Description of the components of a community-based intervention for dengue vector control conducted between June and October 2016 in Ouagadougou, Burkina Faso*

Components	Subcomponents	Activities / By whom	Implemented as intended	Modified	Not implemented	Timeline	Observations
Organization and management	Identification of community-based activities for dengue prevention	Face-to-face household surveys / Researchers	X			May-June 2015	
	Mobilization of community leaders for dengue prevention activities	1 meeting with representatives of the community / Researchers	X			June2016	
	Development of training tools for dengue prevention activities	1 Workshop/ Researchers, representatives of the community, and the theatrical troupe	X			July 2016	
	Training sessions for community group leaders and a theatrical troupe	2 Training sessions / Researchers	X			July 2016	
Communication and education activities	Community campaign	1266 Door-to-door education visits, identification and destruction of *Aedes aegypti* breeding sites / Community members	X			July-October 2016	
		120 Group specific meetings / Community members		X		July-October 2016	Meeting with different socio-economic groups were planned but could not be organized. Meeting were held with small groups (2 to 5 people) gathered in the neighborhood.
		8 Theatrical performances/ Theatrical troupe	X			July-October 2016	

Components	Subcomponents	Activities / By whom	Implemented as intended	Modified	Not implemented	Timeline	Observations
		4 Community activities (public space clean-up activities)	X			August-October 2016	
		A school education session in 4 schools and 11 classrooms (in each classroom) / Researchers and community members	X			September-October 2016	
	Self-awareness assessment	Student drawing competition (755 drawings received) / Researchers and community members		X		September-October 2016	The student drawing competition had not been planned, but was implemented to assess whether they had assimilated the subject content.
	Information	22 Posters displayed for 1 to 7 weeks in public spaces such as schools, places of worships/ Researchers, community members and designer	X			July-October 2016	
		Short message texting / Researchers, community members and mobile operators			X		Short message texts could not be sent because it was difficult for the mobile operators to target the intervention group.
Evaluation	Mid-stream evaluation by researchers	Meetings and on-site visits / Researchers	X			November 2016	
	Mid-stream evaluation by a peer review evaluation committee (community leaders)	Meetings and on-site visits / Community representatives	X			November 2016	

*Summarized from: Sare D, Pérez D., Somé P-A, Kafando Y, Barro A, Ridde V. Community-based dengue control intervention in Ouagadougou: intervention theory and implementation fidelity. Global Health Research and Policy. 2018;3:21. https://doi.org/10.1186/s41256-018-0078-7

Technical Appendix Table 2. Intervention (Tampouy) and control (Juvenat) neighborhood characteristics before and after the community-based intervention for dengue vector control conducted between June and October 2016 in Ouagadougou, Burkina Faso*

	Baseline data		End-line data	
Characteristics	Intervention	Control	Intervention	Control
House Characteristics				
Number of households per compound (mean(SD) /median[range])	1.67(1.22) / 1[1–7]	1.90(1.65) / 1[1–10]	1.38(0.98) / 1[0–6]	1.93(1.83) / 1[0–10]
Number of sets of bedding per compound (mean(SD*) / median[range])	5.62(2.38) / 6[2–12]	6.53(3.57) /.6[1–20]	5.58(2.44) / 5[0–16]	7.11(3.56) / 7[0–23]
Number of residents per compound (mean(SD) / median[range])	8.45(3.98) / 8[1–21]	9.63(5.86) / 10[1–32]	7.43(3.98) / 7[0–22]	9.80(5.52) / 9[0–36]
Characteristics of the respondent to the households' questionnaire				
Head of the household no (%	31(17.61%)	88(54.66%)	37(22.16%)	73(50.34%)
Lady of the household no (%)	108(61.36%)	47(29.19%)	98(58.68%)	43(29.66%)
Female no (/%)	130(75.14%)	78(49.37%)	124(74.70%)	76(52.78%)
Respondent who reported that they “can read”, no (%)	108(62.79%)	133(84.71%)	118(70.66%)	114(78.62%)

*Denominators are different for the variables because missing data were removed before the description.

Technical Appendix Table 3. Impact of the community-based intervention for dengue vector control on primary and secondary outcomes in both intervention and control neighborhoods in Ouagadougou (Differences (β coefficients) for continuous variables and Risk Ratio for binary variables), Burkina Faso

	Baseline data		End-line data		Effect of the intervention	
Variables	Intervention	Control	Intervention	Control	Differences	95% CI
Primary outcomes measured at house level						
Immunological markers (no / Mean (SD))						
∆OD* Nterm-34 kDa	136 / 0.17 (0.10)	136/ 0.13 (0.06)	153 / 0.18 (0.08)	152 / 0.20 (0.12)	−0.08	−0.11; −0.04
Entomological outcomes (no / Mean (SD))						
Number of Ae. Aegypti water breeding sites	184 / 2.65 (2.60)	167 / 1.38 (1.46)	184 / 3.74 (3.22)	167 / 2.27 (2.41)	−0.11	−0.82; 0.61
Number of containers with larvae and/or pupae	184 / 0.29 (0.61)	167 / 0.48 (0.91)	184 / 0.46 (0.71)	167 / 0.66 (1.02)	0.02	−0.21; 0.26
Number of Ae. Aegypti larvae	184 / 9.30 (27.51)	167 / 17.62 (41.52)	184 / 23.73 (49.85)	167 / 23.81 (52.72)	−6.66	−18.40; 5.08
Number of Ae. Aegypti pupae	184 / 1.02 (4.06)	167 / 1.93 (10·82)	184 / 1.81 (7·39)	167 / 2.66 (7·40)	−0.81	−3.40; 1.77
Entomological Indices** (%)						
House index (%)	32.04	33.00	21.36	31.53		
Container index (%)	17.56	30.41	14.43	35.91		
Breteau index (per 100 households)	40.77	54.19	27.67	48.28		
Pupae index (per 100 compounds)	162.14	218.72	99.03	255.67		
Secondary outcomes measured at household level with the questionnaire					RR	95% CI
Knowledge of dengue no (%)						
Heard about dengue	133 (75.57)	130 (80.75)	159 (90.34)	137 (85.09)	1·13	1.01; 1.27
Dengue is a form of malaria	107 (60.80)	99 (61.49)	97 (55.11)	111 (68.94)	0·70	0.58; 0.84
Dengue is dangerous	132 (75.00)	127 (78.88)	153 (86.93)	137 (85.09)	0·99	0.96; 1.02
Fever is a dengue symptom	88 (50.00)	115 (71.43)	127 (72.16)	112 (69.57)	1·44	1.22; 1.69
Dengue is transmitted by a different mosquito than malaria	3 (1.70)	19 (11.80)	88 (50.00)	34 (21.12)	15·33	7.95; 29.55
Attitude and practices against mosquitoes no (%)						
Take measures against mosquitoes	156 (88.64)	142 (88.20)	159 (90.34)	100 (62.11)	1.42	1.29; 1.57
Cover water containers	107 (60.80)	121 (75.16)	127(72.16)	134 (83.23)	1.02	0.91; 1.14
Empty water containers	81 (46.02)	46 (28.57)	117 (66.48)	109 (67.70)	0.58	0.47; 0.72
Use window and door mosquito nets	26 (14.77)	35 (21.74)	22 (12.50)	39 (24.22)	0.74	0.34; 1.58
Use insecticide-treated curtains	11 (6.25)	22 (13.66)	2 (1.14)	12 (7.45)	0.30	0.06; 1.51
Indoor spraying	6 (3.41)	3 (1.86)	10 (5.68)	22 (13.66)	0.24	0.06; 1.00
Clean the house	95 (53.98)	69 (42.86)	156 (88.64)	102 (63.35)	1.09	0.91; 1.29
Have at least one bed-net per 2 residents	100 (56.82)	108 (67.08)	120 (68.18)	98 (60.87)	1.31	1.22; 1.42

*∆OD = level of immunoglobulin G antibody to Nterm-34 kDa peptide in residents' blood spots expressed as ∆OD values
** Entomological Indices were generated at the neighborhood-level and were not modeled with the propensity score.

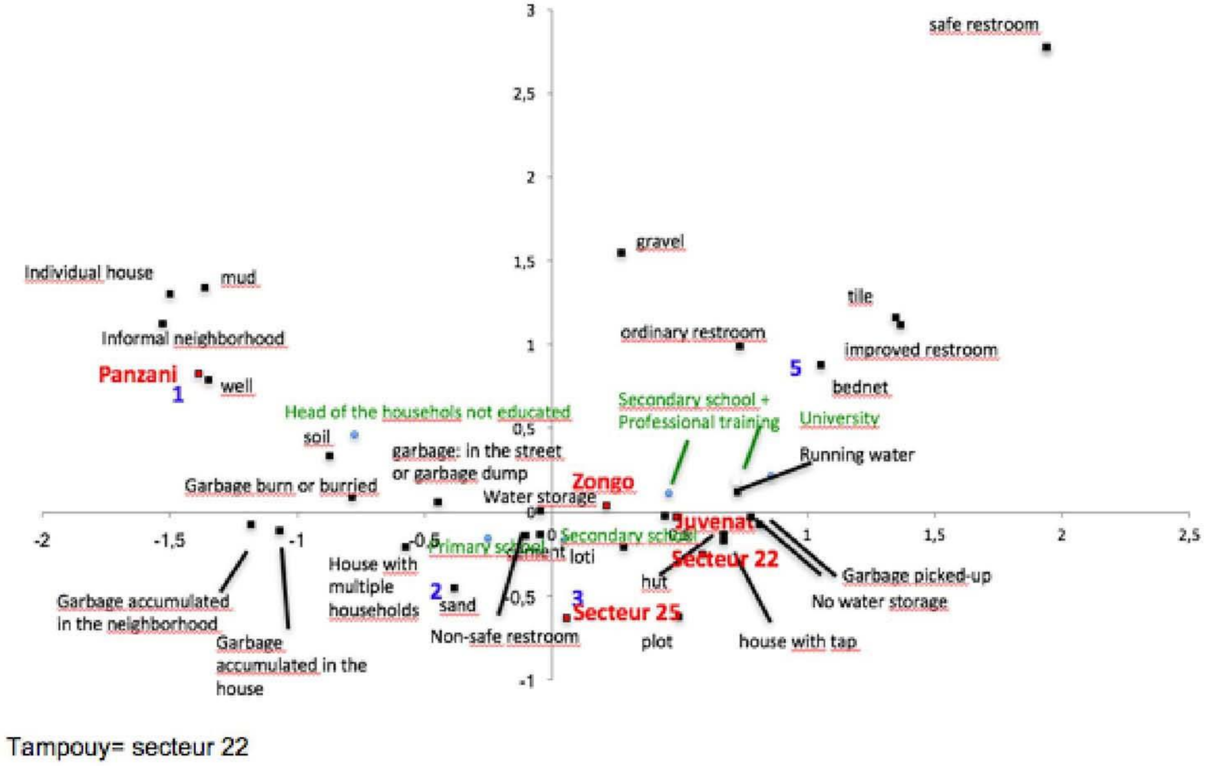

Technical Appendix Figure 1. Distribution and association of household socio-economic characteristics, household education, behaviors, hygiene habits, and exposure to mosquitos in five neighborhoods of Ouagadougou, Burkina Faso.

Primary outcomes		Secondary outcomes
Immunological markers	***Serological data***	
Number of households and sets of bedding in the compound		Wealth Index
Number of residents in the compound		Status of the respondent to the questionnaire
		Household respondent gender
		The respondent self-reported reading ability (cannot read; can read; can read with difficulties)
	For serological data only	
	Status of the individual that provided the blood sample (adult or child)	

Technical Appendix Figure 2. Variables used to generate the Propensity Scores for the primary and secondary outcomes.

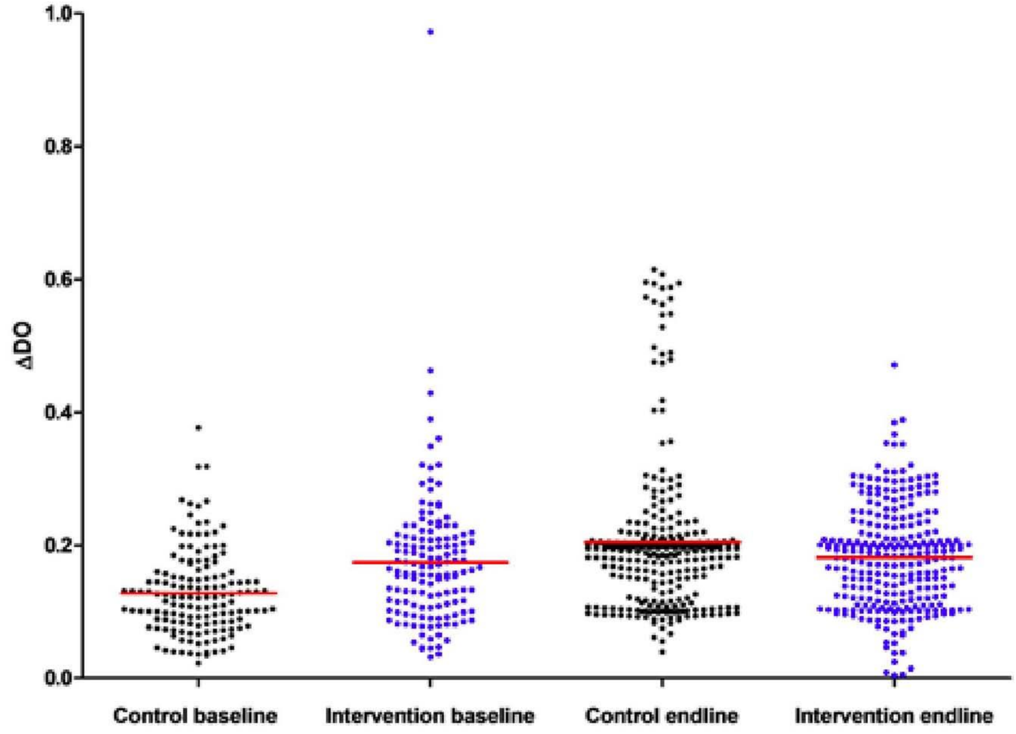

The red lines show the median level of ΔOD.

Technical Appendix Figure 3. Individual IgG response to Nterm-34 kDa peptide in the control and intervention neighborhoods at baseline and end-line.

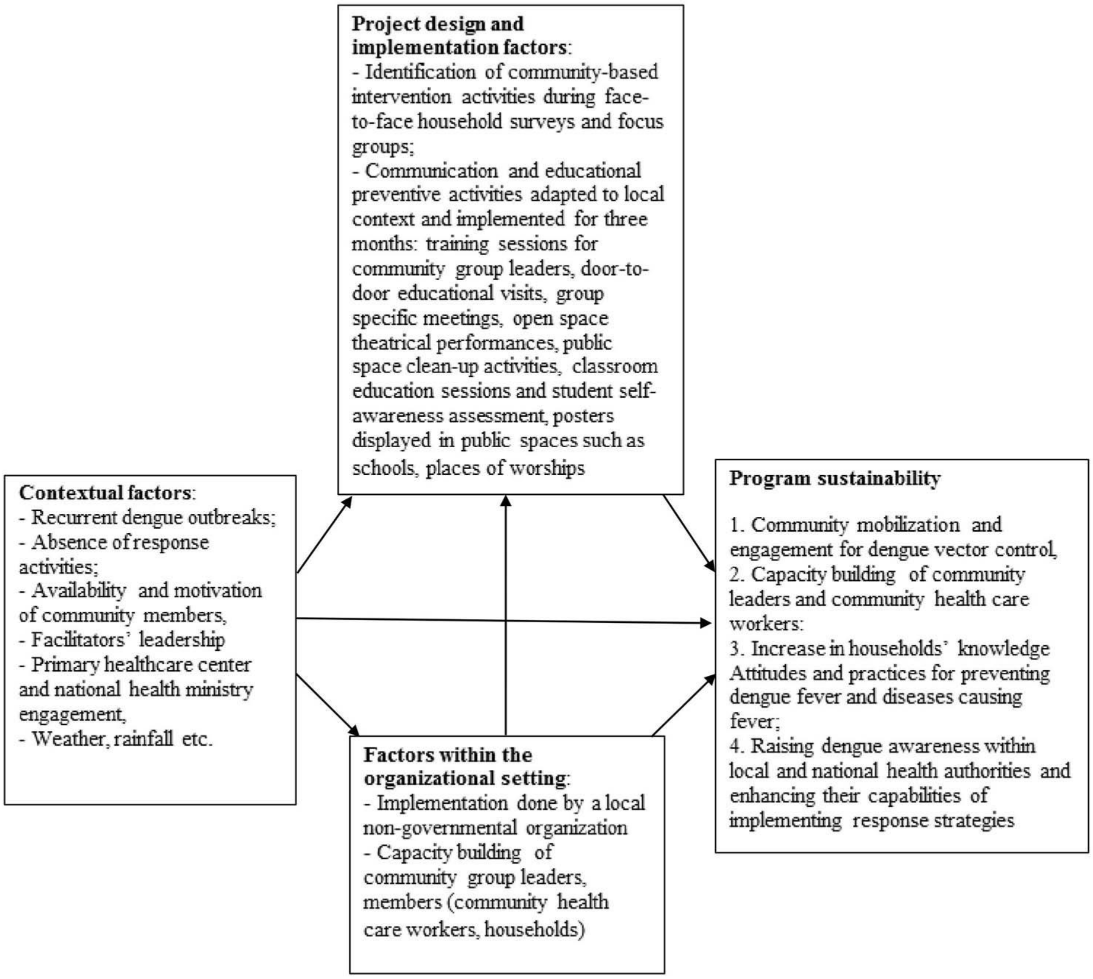

Technical Appendix Figure 4. Sustainability approach to creating the communication materials used in community-based intervention for dengue vector control conducted between June and October 2016 in Ouagadougou, Burkina Faso. Adapted from Shediac-Rizkallah and Bone (1998) sustainability framework.
